# Optical Diffraction in Close Proximity to Plane Apertures. II. Comparison of Half-Plane Diffraction Theories

**DOI:** 10.6028/jres.108.006

**Published:** 2003-02-01

**Authors:** Klaus D. Mielenz

**Affiliations:** National Institute of Standards and Technology, Gaithersburg, MD 20899-8440

**Keywords:** bidirectional fields, diffraction, half plane, irradiance, Kirchhoff, Maxwell, metallic screen, near zone, optics, Poynting, Rayleigh, scalar wave functions, Sommerfeld, wave equation

## Abstract

The accuracy and physical significance of the classical Rayleigh-Sommerfeld and Kirchhoff diffraction integrals are assessed in the context of Sommerfeld’s rigorous theory of half-plane diffraction and Maxwell’s equations. It is shown that the Rayleigh-Sommerfeld integrals are in satisfactory agreement with Sommerfeld’s theory in most of the positive near zone, except at sub-wavelength distances from the screen. On account of the bidirectional nature of diffraction by metallic screens the Rayleigh-Sommerfeld integrals themselves cannot be used for irradiance calculations, but must first be resolved into their forward and reverse components and it is found that Kirchhoff’s integral is the appropriate measure of the forward irradiance. Because of the inadequate boundary conditions assumed in their derivation the Rayleigh-Sommerfeld and Kirchhoff integrals do not correctly describe the flow of energy through the aperture.

## 1. Introduction

In a previous paper [1] this author derived mathematically rigorous expressions for the classical Rayleigh-Sommerfeld and Kirchhoff boundary-value diffraction integrals pertaining to circular apertures and slits illuminated by normally incident plane waves. In spite of their functional differences, these diffraction integrals were found to be surprisingly similar and nearly indistinguishable in most of the near zone. They exhibited significant differences only in the immediate proximity of the aperture, but in this region their physical properties were obscured by the fact that they or their normal derivatives, or both, do not reproduce the assumed incident field. In these circumstances it was not possible to assess their physical significance by merely comparing them to one another. In the present paper, they will be re-examined by applying them to the specific case of diffraction by a reflecting half plane and their physical properties will be interpreted in the context of Sommerfeld’s [2] rigorous theory of half-plane diffraction and Maxwell’s equations.

## 2. Comparison of Scalar Wave Functions

**Fig. A1:**
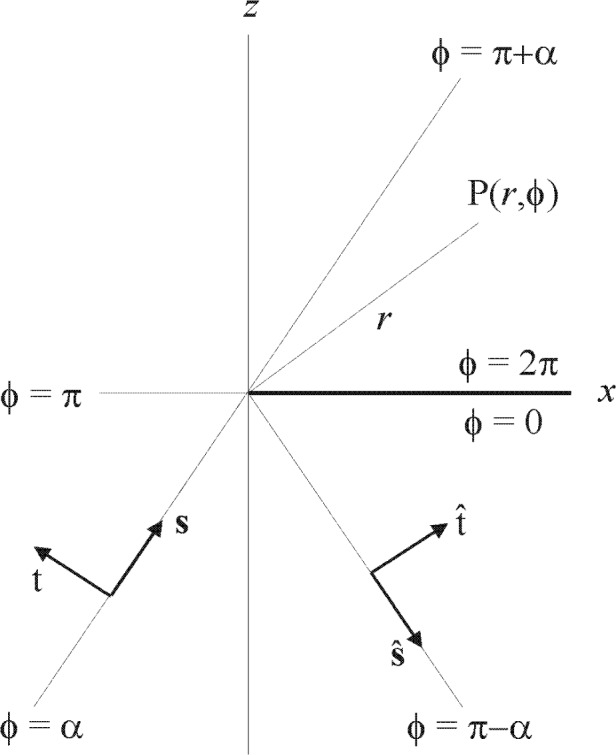
Basic geometry and notation for Sommerfeld’s theory.

The scalar wave functions *U* discussed in this paper all denote the complex disturbance at a point of observation P(*x*, *y*, *z*) in the diffraction pattern of a perfectly conducting, infinitesimally thin, semi-infinite screen that occupies the half plane *x* > 0, *z* = 0 of a cartesian coordinate system, as depicted in Fig. A1 of Appendix A. The primary field is assumed to be a monochromatic plane wave with irradiance *E*_0_, wavelength *λ*, and circular wave number *k* = 2π/*λ* that is normally incident from the half space *z* < 0 and is plane polarized so that, in accordance with Maxwell’s equations, ∂*U*/∂z or *U* are continuous and equal to zero on crossing the screen. The resulting diffraction pattern is independent of *y* and will be denoted by $U=\sqrt{E_{0}}u\left(x,\;z\right)$, so that we have |*u*| ≤ 1.

Sommerfeld’s half-plane theory dates back to the late 1800s and used to be discussed at length in textbooks [3–5]. However, it appears to be no longer included in modern curricula of theoretical optics, and therefore its main features are summarized and supplemented by new expressions for the diffracted irradiance in Appendix A, below. On combining Eqs. (A3a–c) and (A8a,b) of Appendix A it follows that, for normally incident light, Sommerfeld’s solution is reduced to

$$\begin{array}{c} u_{S}^{\left(p,s\right)}\left(x,\;z\right)=u_{S}\mp {\hat{u}}_{S},\\ u_{S}=e^{ikz}V(\rho ),\;{\hat{u}}_{S}=e^{-ikz}V(\hat{\rho }), \end{array}$$ (1a)

$$\rho =\sqrt{\frac{2}{\lambda }}[-\text{sign}(z)\sqrt{r+x}-\sqrt{r-x}],\;z\neq 0,$$ (1b)

$$\hat{\rho }=\sqrt{\frac{2}{\lambda }}[-\text{sign}(z)\sqrt{r+x}+\sqrt{r-x}],\;z\neq 0,$$ (1c)

where $r=\sqrt{r^{2}+z^{2}}$ and V(*ρ*) is the complex Fresnel-type integral defined by Eq. (A3d). These expressions are rigorously valid everywhere in the *xz*-plane of Fig. A1, except that along the *x*-axis *ρ* and $\hat{\rho }$ must be evaluated as

$$\rho =2\sqrt{\frac{\left|x\right|}{\lambda }},\;\hat{\rho }=2\text{sign}(x)\sqrt{\frac{\left|x\right|}{\lambda }},\;z=-0,$$ (1d)

$$\rho =-2\text{sign}(x)\sqrt{\frac{\left|x\right|}{\lambda }},\;\hat{\rho }=-2\sqrt{\frac{\left|x\right|}{\lambda }},\;z=+0,$$ (1e)

where z = ±0 refers to the positive and negative sides of the screen, respectively. This distinction is necessary because $u_{S}^{\left(p\right)}$ and $\partial u_{S}^{\left(s\right)}/\partial z$ are discontinuous on crossing the screen, and is taken into account in Sommerfeld’s theory by “wrapping” the diffracting half plane in a semi-infinite, two-sided Riemann surface so that its positive and negative sides are distinguished by the values 2π and 0 of the polar angle *ϕ* in Fig. A1.

The corresponding results given by the Rayleigh-Sommerfeld theory are obtained from Eqs. (10a,b) of Ref. [1] by suitably modifying the limits of integration, leading to

$$\begin{array}{c} u_{\text{RS}}^{\left(p\right)}(x,\;z)=\frac{k}{2}\int_{-\infty }^{-x}d(\xi -x)H_{0}^{\left(1\right)}(\beta ),\\ \beta =k\sqrt{{\left(\xi -x\right)}^{2}+z^{2}},\;z>0, \end{array}$$ (2a)

$$u_{\text{RS}}^{\left(s\right)}(x,\;z)=\frac{ik^{2}z}{2}\int_{-\infty }^{-x}d(\xi -x)\frac{H_{1}^{\left(1\right)}(\beta )}{\beta },\;z>0,$$ (2b)

where $H_{n}^{\left(1\right)}-J_{n}+{\text{iY}}_{n}$ are Hankel functions of the first kind and *n*th order. These expressions are valid for *z* > 0, only, and will be supplemented in this paper by the assumptions made in their derivation for *z* ≤ 0; namely,

$$\begin{array}{c} \frac{\partial u_{\text{RS}}^{\left(p\right)}(x,\;+0)}{\partial z}=ik\;\text{or}\;0,\;u_{\text{RS}}^{\left(s\right)}(x,+0)=1\;\text{or}\;0,\\ x<0\;\text{or}\;x\geq 0,\;\text{for}\;z=+0, \end{array}$$ (2c)

and

$$\begin{array}{c} u_{\text{RS}}^{\left(p,s\right)}(x,\;z)=e^{ikz}\;\text{or}\;(e^{ikz}\mp e^{-ikz}),\\ x<0\;\text{or}\;x\geq 0,\;\text{for}\;z\leq -0 \end{array}$$ (2d)

respectively.

Kirchhoff’s diffraction integral, which will be required for the discussion in Sec. 3 is equal to the arithmetic mean of the Rayleigh-Sommerfeld integrals (2a,b),

$$u_{K}(x,\;z)=\frac{1}{2}[u_{\text{RS}}^{\left(p\right)}(x,\;z)+u_{\text{RS}}^{\left(s\right)}(x,\;z)],$$ (2e)

and can therefore be easily deduced from the above expressions.

In the paraxial Fresnel approximation where *z* is positive and large compared to *λ* and *x*/*z* is small all of the above-mentioned solutions converge to the familiar Fresnel limit *u*_F_. That is,

$$\begin{array}{c} u_{S}^{\left(p,s\right)}(x,\;z)∼u_{\text{RS}}^{\left(p,s\right)}(x,\;z)∼u_{K}(x,\;z)∼u_{F}(x,\;z)\\ =e^{ikz}V(-x\sqrt{\frac{2}{\lambda z}}),\;z>>\left|x\right|, \end{array}$$ (3a)

where the right-hand expression follows from Eqs. (1a–c) by letting $\sqrt{r\pm x}∼\sqrt{z}\;[1\pm x/(2z)]$, so that $\rho =-x\sqrt{2/\lambda z}$, $\hat{\rho }=-\sqrt{8z/\lambda }$, and ${\hat{u}}_{S}<<u_{S}$.^1^ The same result is obtained from Eqs. (2a,b) on replacing $H_{0}^{\left(1\right)}$, and $H_{1}^{\left(1\right)}$ and *β* by the leading terms of their asymptotic and Taylor expansions. The Fresnel approximation, Eq. (3a), is estimated to be accurate within 1 % for *z* >> 100*λ*.

For numerical applications it is also useful to know that the above solutions all predict the same value,

$$\begin{array}{c} u_{S}^{\left(p,s\right)}(0,\;z)=u_{\text{RS}}^{\left(p,s\right)}(0,\;z)=u_{K}(0,\;z)\\ =u_{F}(0,\;z)=\frac{1}{2}e^{ikz}, \end{array}$$ (3b)

in the positive shadow boundary (x = 0, *z* > 0). In the case of the Rayleigh-Sommerfeld integrals, Eqs. (2a,b), this result follows from the identity

$$\int_{0}^{\infty }dtH_{0}^{\left(1\right)}(\sqrt{a^{2}+t^{2}})=e^{ia},$$ (3c)

and was used in this work as the starting value for recursive numerical integrations as described in Ref. [6].

**Fig. 1:**
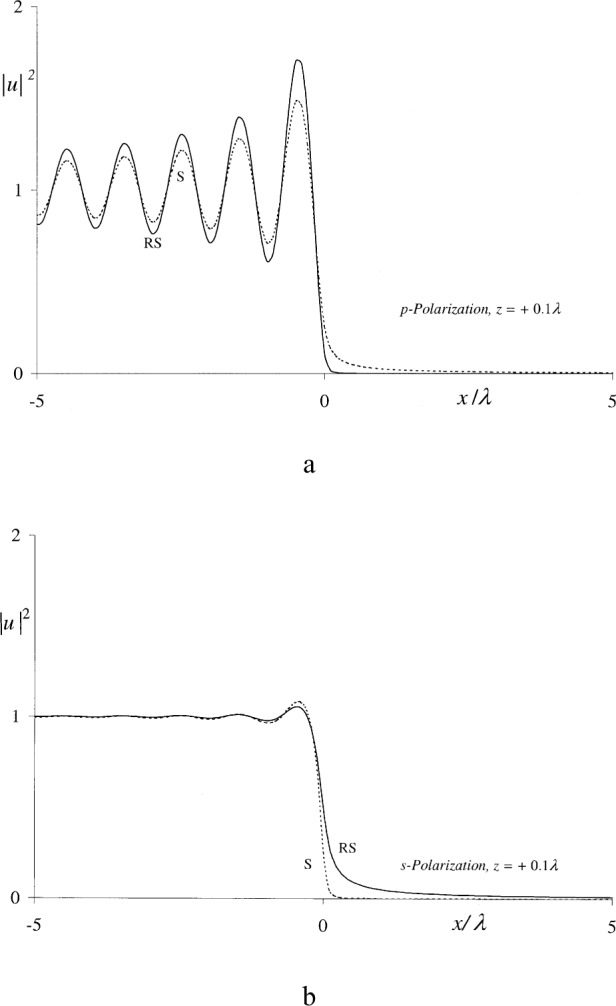
${\left|u_{S}^{\left(p,s\right)}(x,\;z)\right|}^{2}$ (-----) and ${\left|u_{\text{RS}}^{\left(p,s\right)}(x,\;z)\right|}^{2}$ (–—) vs *x*/*λ* at the distance *z* = +0.1*λ* from the aperture plane.

**Fig. 2:**
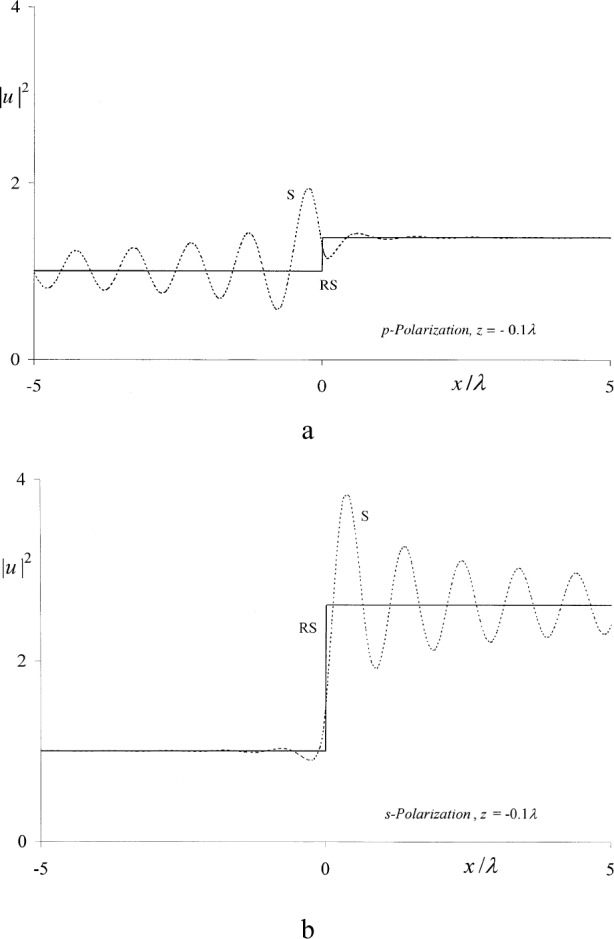
${\left|u_{S}^{\left(p,s\right)}(x,\;z)\right|}^{2}$ (-----) and ${\left|u_{\text{RS}}^{\left(p,s\right)}(x,\;z)\right|}^{2}$ (–—) vs *x*/*λ* at the distance *z* = –0.1*λ* from the aperture plane.

The above expressions for $u_{S}^{\left(p,s\right)}(x,\;z)$ and $u_{\text{RS}}^{\left(p,s\right)}(x,\;z)$ were used to compute the squared magnitudes of these functions in the immediate proximity of the positive and negative sides of the aperture plane, as shown in Figs. 1 and 2. For these computations, Eqs. (2a,b) were evaluated as noted above and the Fresnel sine and cosine integrals required for the computation of V(*ρ*) and $V\left(\dot{\rho }\right)$ were evaluated using the algorithms of Ref. [7]. The main conclusions drawn from these results are as follows.

1. On the positive side of the aperture plane the Sommerfeld and Rayleigh-Sommerfeld solutions are surprisingly similar, even at very small distances *z*. The real and imaginary parts of $u_{S}^{\left(p,s\right)}$ and $u_{\text{RS}}^{\left(p,s\right)}$ contributing to the results plotted in Fig. 1 agree within ± 1 % or better for *z* = 0.1*λ*, and additional computations showed that this agreement improves rapidly for larger values of *z*. It follows that for all practical purposes the Rayleigh-Sommerfeld integrals are adequate for computations throughout the positive near zone, and hence it may be inferred that this will also be the case for the corresponding solutions for circular apertures and slits derived in Ref. [1].
2. The agreement for negative values of *z* is unsatisfactory. In Sommerfeld’s theory diffraction manifests itself as a field phenomenon that occurs on both sides of the aperture plane, so that the incident geometrical field is modified before it reaches the screen. On the other hand, in the Rayleigh-Sommerfeld theory diffraction on the source side is explicitly ruled out, and here the results obtained from Sommerfeld’s theory show that the assumed geometrical field (2d) is only a crude approximation of the true field. Thus, the main problem with the Rayleigh-Sommerfeld and Kirchhoff integrals appears to be not so much that they fail to reproduce the assumed geometrical field values, but that the latter are themselves objectionable.

The residual differences between $u_{\text{RS}}^{\left(p,s\right)}$ and $u_{S}^{\left(p,s\right)}$ for *z* > 0 can be attributed to the imperfect boundary conditions assumed in the Rayleigh-Sommerfeld theory. These boundary values are step functions that violate the wave equation and are the probable cause of the fact, shown in Appendix B, that the Rayleigh-Sommerfeld integrals also do not obey the wave equation in the immediate proximity of the aperture plane. Although this wave-equation failure is small in most of the near zone, and thus unimportant for practical purposes, it is worthwhile to mention that it might be remedied by replacing the boundary values Eq. (2c) with the corresponding values given by Sommerfeld’s theory for *z* = +0; namely,

$$\begin{array}{c} \frac{1}{ik}\frac{\partial u_{S}^{\left(p\right)}(x,+0)}{\partial z}=1\;\text{or}\;2V(-2\sqrt{\frac{x}{\lambda }})-\frac{i}{ð}\sqrt{\frac{\lambda }{2x}}e^{i(kx-\frac{ð}{4})},\\ x<0\;\text{or}\;x\geq 0, \end{array}$$ (4a)

$$u_{S}^{\left(s\right)}\left(x,+0\right)=1\;\text{or}\;2V\left(-2\sqrt{\frac{x}{\lambda }}\right),x<0\;\text{or}\;x\geq 0.$$ (4b)

**Fig. 3:**
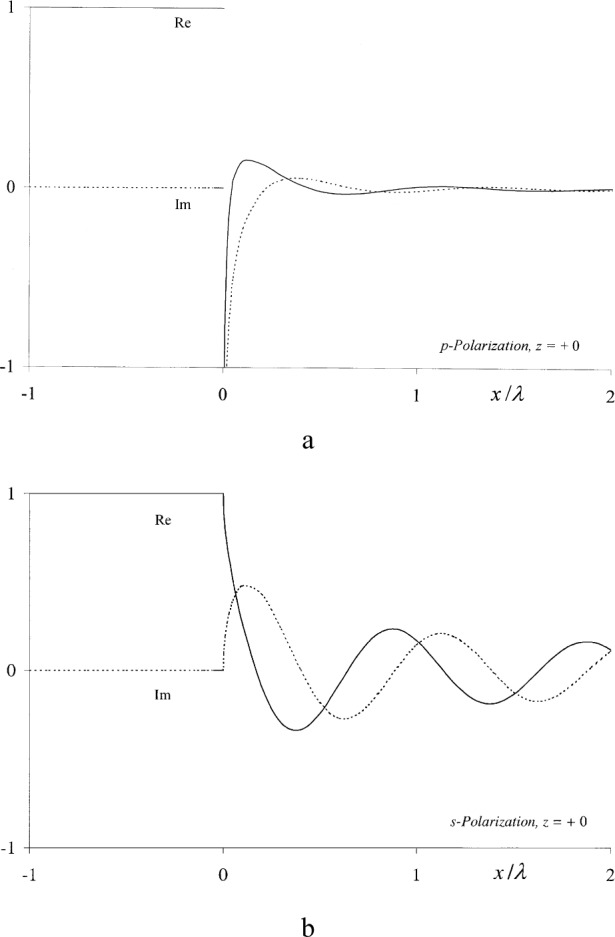
Real (–—) and imaginary (-----) parts of the boundary values Eqs. (4a,b) predicted by Sommerfeld’s theory for p- and s-polarized lincident light.

The real and imaginary parts of these functions are plotted in Fig. 3, where it should be noted that $\partial u_{S}^{\left(p\right)}/\partial z$ is discontinuous and singular, and $u_{S}^{\left(s\right)}$ is not continuously differentiable, for *x* = 0. Nonetheless, they constitute improved boundary values because Sommerfeld’s theory obeys the wave equation even at the diffracting edge itself (see Appendix B).

When Eqs. (4a,b) are substituted into the derivation of the Rayleigh-Sommerfeld integrals for the half plane one finds

$$u_{S}^{\left(p\right)}(x,\;z)\equiv \frac{k}{2}\int_{-\infty }^{\infty }d(\xi -x)\frac{\partial u_{S}^{\left(p\right)}(x,+0)}{\partial z}H_{0}^{\left(1\right)}(\beta ),\;z\geq 0,$$ (4c)

$$u_{S}^{\left(p\right)}(x,\;z)\equiv \frac{ik^{2}z}{2}\int_{-\infty }^{\infty }d(\xi -x)u_{S}^{\left(p\right)}(x,+0)\frac{H_{1}^{\left(1\right)}(\beta )}{\beta },\;z\geq 0,$$ (4d)

where the integration now extends from –∞ to +∞. Because the boundary values Eq. (2c) and Eqs. (4a,b) are the same for *x* < 0 and the former are zero for *x* ≥ 0, this can be rewritten as

$$u_{S}^{\left(p,\;z\right)}(x,\;z)\equiv u_{\text{RS}}^{\left(p,\;z\right)}(x,\;z)+\Delta u_{\text{RS}}^{\left(p,\;z\right)},$$ (4e)

where

$$\begin{array}{c} \Delta u_{\text{RS}}^{\left(p\right)}=u_{S}^{\left(p\right)}-u_{\text{RS}}^{\left(p\right)}\\ =k\int_{-x}^{\infty }d(\xi -x)[V(-2\sqrt{\frac{x}{\lambda }})-\frac{i}{2ð}\sqrt{\frac{\lambda }{2x}e^{i(kx-\frac{ð}{4})}}]H_{0}^{\left(1\right)}(\beta ), \end{array}$$ (4f)

$$\Delta u_{\text{RS}}^{\left(s\right)}=u_{S}^{\left(s\right)}-u_{\text{RS}}^{\left(s\right)}=ik^{2}z\int_{-x}^{\infty }d(\xi -x)V(-2\sqrt{\frac{x}{\lambda }})\frac{H_{1}^{\left(1\right)}(\beta )}{\beta }$$ (4g)

are correction terms that can be added to the Rayleigh-Sommerfeld integrals to convert them to the exact values given by Sommerfeld’s theory. These expressions should be free of errors because Eqs. (4c) and (4d) are rigorous expressions of the Helmholtz’ theorem in which $u_{S}^{\left(p\right)}$ and $u_{S}^{\left(s\right)}$ are the same on both sides of the equal sign.

This method was originally proposed by Braunbek [8–10], who envisioned its use for constructing improved solutions for large apertures of finite width and are bounded by straight or even curved edges. Braunbek’s work involved the assumption that $\partial u_{S}^{\left(p\right)}/\partial z$ and $u_{S}^{\left(s\right)}$ rapidly become negligibly small on the dark side of the screen, so that the effective ranges of integration in Eqs. (4f,g) are only a few wavelengths wide and approximative methods can be used. According to Fig. 3 this is a valid assumption for $\partial u_{S}^{\left(p\right)}/\partial z$ but not for $u_{S}^{\left(s\right)}$, so that computational difficulties could be encountered in the case of $\Delta u_{\text{RS}}^{\left(s\right)}$.

## 3. Irradiance and Energy Flow

Although the squared magnitudes of scalar wave functions are commonly identified with the irradiance of the field, the data plotted in Figs. 1 and 2 must not be interpreted in this manner. The diffracted field specified by Sommerfeld’s solution is a bidirectional field composed of two plane waves, *u*_S_ and ±*û*_S_ which propagate in the opposite directions of the incident primary field and its reflection from the screen. When Maxwell’s equations are invoked, as in Eqs. (A5) through (A7) of Appendix A, it is found that in accordance with the principle of interference these waves cannot interfere with one another^2^ so that the effective energy flow is composed of mutually incoherent components in the forward and reverse directions. For normally incident light, these respective directions are parallel and anti-parallel to the unit vector ***n*** = [0,0,1] in the direction of the positive *z*-axis, and the final expression for the time-averaged Poynting vector (A7c) is

$$<S_{S}>=[E_{S}(x,\;z)-{\hat{E}}_{S}(x,\;z)]n=E_{0}({\left|u_{S}\right|}^{2}-{\left|{\hat{u}}_{S}\right|}^{2})n,$$ (5)

where *E*_S_ and *Ê*_S_ are the forward and reverse irradiances incident on the opposite sides of any given area element d*x* d*y* containing the point of observation P.^3^ These irradiances are given by the squared magnitudes of the basic Sommerfeld functions *u*_S_ and *û*_S_ themselves, and thus the quantities |*u*_S_ − *û*_S_|^2^ or |*u*_S_ + *û*_S_|^2^ do not represent the irradiances of the field for *p*- and *s*-polarized light. Accordingly, the forward and reverse irradiances of the field are independent of the state of polarization of the incident light, and in this connection it should also be noted that in practice the reverse irradiance *Ê*_S_ is not easily observable as it may be obscured by a detector placed in the path of the forward field.

It now seems reasonable to interpret the Rayleigh-Sommerfeld theory in a like manner, so that the quantities $u_{\text{RS}}^{\left(p\right)}$ and $u_{\text{RS}}^{\left(s\right)}$ defined by Eqs. (2a,b) are also regarded as bidirectional wave functions that can be resolved into mutually incoherent forward and reverse components, *u*_K_ and *û*_K_. Thus we define, in analogy to Eq. (1a),

$$\begin{array}{l} u_{K}=\frac{1}{2}[u_{\text{RS}}^{\left(p\right)}(x,\;z)+u_{\text{RS}}^{\left(s\right)}(x,\;z)],\\ {\hat{u}}_{K}=\frac{1}{2}[u_{\text{RS}}^{\left(p\right)}(x,\;z)-u_{\text{RS}}^{\left(s\right)}(x,\;z)]. \end{array}$$ (6a)

and hence it follows that the corresponding forward and reverse irradiances, *E*_K_ and *Ê*_K_, will be given by an expression analogous to Eq. (5),

$$<S_{K}>=[E_{K}(x,\;z)-{\hat{E}}_{K}(x,\;z)]n=E_{0}({\left|u_{K}\right|}^{2}-{\left|{\hat{u}}_{K}\right|}^{2})n.$$ (6b)

It will be noted that the forward wave function *u*_K_ defined by Eq. (6a) and Kirchhoff’s integral (2e) are identically the same, and therefore the subscript “K” was retained in the above equations. The Kirchhoff and Rayleigh-Sommerfeld solutions were originally derived on the mutually exclusive assumptions of black and metallic screens, and it is generally agreed that Eq. (2c) has no definable physical meaning as it would somehow imply the coherent superposition of two orthogonal states of polarization. However, in the present context, the Rayleigh-Sommerfeld integrals are interpreted as composite quantities and taking their sum and difference is tantamount to resolving them into their basic components. Accordingly, Kirchhoff’s integral *u*_K_ now appears as an integral part of the Rayleigh-Sommerfeld theory for metallic screens so that $u_{\text{RS}}^{\left(p\right)}$ and $u_{\text{RS}}^{\left(s\right)}$ provide the framework for the evaluation of all field parameters while *u*_K_ and its counterpart *û*_K_ define the flow of field energy. This new interpretation of Kirchhoff’s integral has a precise, physically realizable meaning.

**Fig. 4:**
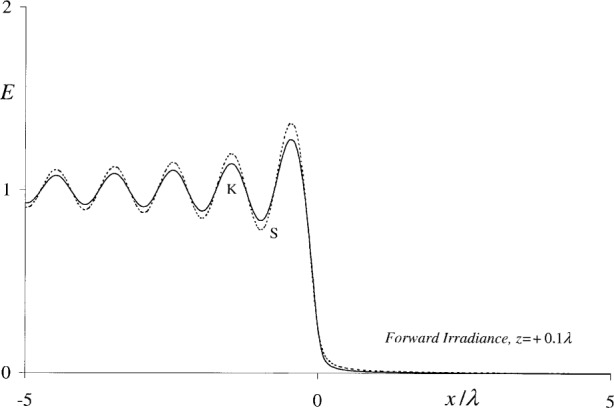
Forward irradiances *E*_S_(*x*,*z*) (-----) and *E*_K_(*x*,*z*) (–—) vs *x*/*λ* at the distance *z* = +0.1*λ* from the aperture plane.

**Fig. 5:**
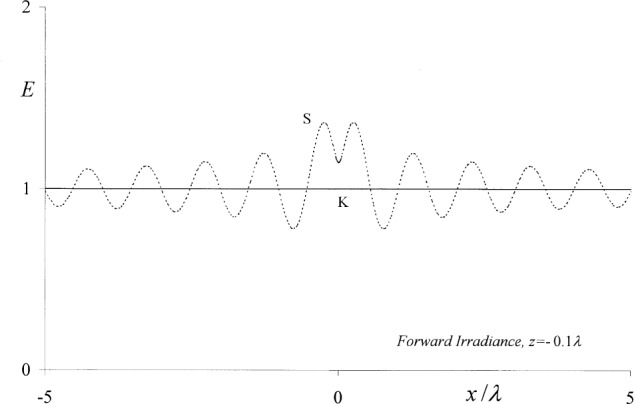
Forward irradiances *E*_S_(*x*,*z*) (-----) and *E*_K_(*x*,*z*) (–—) vs *x*/*λ* at the distance *z* = –0.1*λ* from the aperture plane.

A numerical comparison of the forward irradiances *E*_S_ and *E*_K_ defined by Eqs. (5) and (6b) is presented in Figs. 4 and 5. As expected, these quantities are essentially the same on the positive side of the aperture plane, the agreement being on the order of a few percent for *z* = +0.1*λ* and increasingly better for larger values of *z*. This confirms that the identification of |*u*_K_|^2^ with the forward irradiance *E*_K_ is a valid assumption. As also expected, the agreement is poor on the negative side because in this region *E*_K_ represents only the undiffracted geometrical field. The even symmetry of the irradiance *E*_s_ shown in Fig. 5 suggests that the modification of the geometrical field due to diffraction is isotropic in the immediate vicinity of the edge.

## 4. Conclusions

The above comparison of the classical Rayleigh-Sommerfeld boundary-value theories with Sommerfeld’s rigorous theory for diffraction by a perfectly reflecting half plane has added substantially to the understanding of the physical significance of these theories.

It was found that the mathematical expressions and algorithms presented in Ref. [1] for the Rayleigh-Sommerfeld integrals are in very satisfactory agreement with Sommerfeld’s half-plane theory. Thus, they are well suited for computations in most of the positive near zone, and it is inferred that this will also be the case for the corresponding Rayleigh-Sommerfeld integrals and slits derived in Ref. [1]. Sommerfeld’s theory also confirms that, on the whole, the differences between these respective solutions for *p*- and *s*-polarized incident light are small so that polarization effects are small, as might be expected for normally incident light. All in all, it appears that the use of Helmholtz’ theorem has proved remarkably effective in compensating for the inadequate boundary conditions assumed in deriving the classical boundary-value integrals. The residual differences between the Rayleigh-Sommerfeld and Sommerfeld solutions are confined to sub-wavelength differences from the screen, and it is shown in Appendix B that in this region the former do not obey the wave equation.

The comparison with Sommerfeld’s theory and its interpretation in terms of Maxwell’s equations has also revealed a previously overlooked aspect of diffraction by a reflecting screen; namely, that the optical field is bidirectional and comprises light traveling in opposite directions even on the positive side of the screen. According to the principle of interference, the observable Poynting vector is given by the incoherent vector sum of its components in the forward and reverse components, and thus it is impermissible to express the near-zone irradiance of the field as the squared magnitudes of scalar wave functions. Rather, the latter must be resolved into their forward and reverse component and it turns out that Kirchhoff’s integral is the appropriate expression for the forward irradiance of the field even in the Rayleigh-Sommerfeld theory. The forward and reverse irradiances were found to be independent of the state of polarization of the incident field.

It was noted that the residual deficiencies of the Rayleigh-Sommerfeld and Kirchhoff solutions in the proximity of the positive aperture plane can be removed by replacing the originally assumed boundary values with those predicted by Sommerfeld’s theory. This was not be pursued further as it would produce only marginal improvements on the positive side of the screen, without removing the problem that the classical boundary-value integrals all exhibit discontinuities with respect to the incident geometrical field. A more effective approach would be the derivation of improved approximations for the entire field by constructing analytical continuations of the existing boundary-value solutions into the half space *z* ≤ 0. This will be attempted in a subsequent publication.

## Figures and Tables

**Fig. A2:**
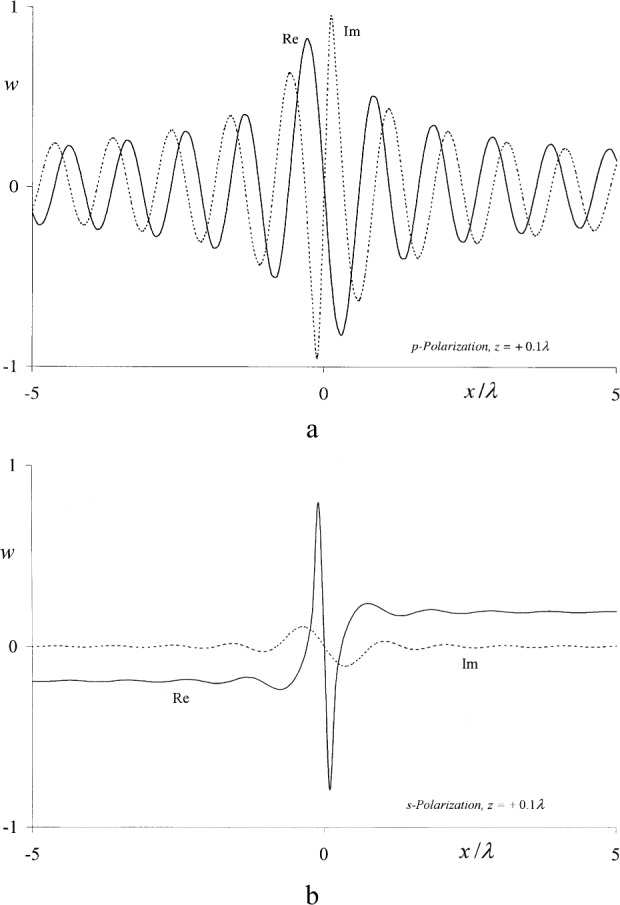
Real (–—) and imaginary (-----) parts of the quantities defined by Eqs. (A10h,i) at the distance *z* = +0.1*λ* from the aperture plane.

## Footnotes

1 For large negative values of *z*, Sommerfeld’s theory also yields a comlementary term for the Fresnel approximation of the reflected diffraction pattern on the lit side of the half plane.

2 Except for grazing incidence, where interference can occur as in Fresnel’s mirror experiment.

3 For oblique incidence of the primary field, as assumed in Eq. (A8a), these irradiances are defined with respect to area elements which are perpendicular to the directions of propagation of the incident and reflected geometrical field.

## 5. Appendix A. Sommerfeld’s Half-Plane Theory

In Sommerfeld’s rigorous treatment of diffraction by a straight edge the screen is assumed to be a perfectly conducting, infinitesimally thin, semi-infinite sheet that covers the half-plane *x* > 0, *z* = 0 of the Cartesian coordinate system in Fig. A1. It is assumed, further, that the primary field is a monochromatic plane wave, *p*- or *s*-polarized with respect to the *xz*-plane and incident upon the screen in a given angular direction *α*. As the optical field so defined must be independent of *y* it will be convenient to use cylindrical coordinates (*r*,*ϕ*,*y*) given by

$$r=\sqrt{x^{2}+z^{2}},\;\phi =\arccos(\frac{x}{r}),\;x=r\cos\phi ,\;z=-r\sin\phi ,$$ (A1a)

so that *ϕ* and *α* are measured clockwise from the positive *x*-axis, and the illuminated and shaded sides of the screen are distinguished by *ϕ* = 0 and 2π, respectively.^4^ In this notation, the primary field, its reflection by the screen, and the unit vectors in their respective directions of propagation are

$$U_{\text{geom}}=\sqrt{E_{0}}e^{-ikr\cos(\phi -\alpha )},{\hat{U}}_{\text{geom}}=\mp \sqrt{E_{0}}e^{-ikr\cos(\phi +\alpha )}.$$ (A1b)

where the time factor of the field is assumed as e^–i^*^ωt^*, *k* = 2π/*λ* is the circular wavenumber of the light, *E*_0_ is the incident irradiance, and the dual sign of *Û*_geom_ accounts for polarization-dependent phase changes on reflection.

According to these definitions, the diffracted field at a given point P(*r*,*ϕ*) must obey the scalar wave equation,

$$\Delta U^{\left(p,s\right)}=\frac{\partial^{2}U^{\left(p,s\right)}}{\partial r^{2}}+\frac{1}{r}\frac{\partial U^{\left(p,s\right)}}{\partial r}+\frac{1}{r^{2}}\frac{\partial^{2}U^{\left(p,s\right)}}{\partial \phi^{2}}=-k^{2}U^{\left(p,s\right)},$$ (A2a)

as well as the boundary conditions for *ϕ* = 0 and *ϕ* = 2π,

$$U^{\left(p\right)}=0\;\text{or}\;\frac{\partial U^{\left(s\right)}}{\partial z}=0,\;\text{for}\;\phi =0\;\text{and}\;2\pi ,$$ (A2b)

according as the light is *p*- or *s*-polarized. Furthermore, in the limit *r*→∞ these solutions must correspond to the optical field according to geometrical optics, and in this respect it is necessary to distinguish three regions of space as indicated in Fig. A1:

- The reflection space (0 < *ϕ* < *α*), where the incident and reflected waves are both present and the geometrical field is $U_{\text{geom}}^{\left(p,s\right)}=U_{\text{geom}}\mp {\hat{U}}_{\text{geom}}$.
- The transmission space (*α* < *ϕ* < *α* + π), where only the incident wave is present and the geometrical field is $U_{\text{geom}}^{\left(p,s\right)}=U_{\text{geom}}$.
- The shadow space (*α* + π < *ϕ* < 2π), where the geometrical field $U_{\text{geom}}^{\left(p,s\right)}$ is zero.

Sommerfeld’s solution of the diffraction problem so defined is:

$$U_{S}^{\left(p,s\right)}(r,\phi )=\sqrt{E_{0}}(u_{S}\mp {\hat{u}}_{S}),$$ (A3a)

$$u_{S}=e^{-ikr\cos(\phi -\alpha )}V(\rho ),\;{\hat{u}}_{S}=e^{-ikr\cos(\phi +\alpha )}V(\hat{\rho }),$$ (A3b)

$$\rho =\sqrt{\frac{8r}{\lambda }}\cos\left[\frac{1}{2}(\phi -\alpha )\right],\;\hat{\rho }=\sqrt{\frac{8r}{\lambda }}\cos\left[\frac{1}{2}(\phi +\alpha )\right]$$ (A3c)

$$\begin{array}{c} V(\rho )=\frac{e^{-i\frac{ð}{4}}}{\sqrt{2}}\int_{-\infty }^{\rho }d\tau e^{\frac{ið\tau^{2}}{2}}=\frac{1}{2}[1=C(\rho )+S(\rho )]\\ -\frac{i}{2}[C(\rho )-S(\rho )], \end{array}$$ (A3d)

where C(*ρ*) and S(*ρ*) denote the usual Fresnel cosine and sine integrals.

Although the derivation of these expressions is too complicated to be included in this paper, it is not difficult to verify that they have the following properties:

1. As shown in Appendix B,*u*_S_ and *û*_S_ obey the wave equation, Eq. (A2a), everywhere in space, inclusive of the diffracting edge itself. They represent plane waves which propagate with space-dependent amplitudes, V(*ρ*) and $V\left(\hat{\rho }\right)$, in the respective directions of the unit vectors, Eq. (A1c). Except in the reflection space and at small distances *r* from the diffracting edge, *û*_S_ is significantly smaller than *u*_S_, and in the limit *r* → ∞ Eqs. (A3a,b) are reduced to the above-mentioned geometrical solutions.
2. In addition to the usual plane-wave components which are proportional to *u*_S_ and *û*_S_ themselves, the derivatives,
  $$\left(\begin{array}{c} \partial u_{S}/\partial x\\ \partial {\hat{u}}_{S}/\partial x \end{array}\right)=-ik\cos\alpha \left(\begin{array}{c} u_{S}\\ {\hat{u}}_{S} \end{array}\right)+\cos\left[\frac{1}{2}(\phi \pm \alpha )\right]\frac{e^{i(kr-\frac{ð}{4})}}{\sqrt{\lambda r}},$$ (A4a)
  $$\left(\begin{array}{c} \partial u_{S}/\partial z\\ \partial {\hat{u}}_{S}/\partial z \end{array}\right)=\pm ik\sin\alpha \left(\begin{array}{c} u_{S}\\ {\hat{u}}_{S} \end{array}\right)-\sin\left[\frac{1}{2}(\phi \pm \alpha )\right]\frac{e^{i(kr-\frac{ð}{4})}}{\sqrt{\lambda r}},$$ (A4b)
  contain terms which involve the derivatives of V(*ρ*) and $V\left(\hat{\rho }\right)$ with respect to *r* and, thus, are singular as $1/\sqrt{r}$ at the diffracting edge. This suggests the existence of cylindrical waves which originate at the edge, but nonetheless the edge does not radiate energy. Although the radiant intensity *I* of the edge is infinite as $1/\sqrt{r}$, the radiant flux *I*d*Ω* emitted into any given solid angle element d*Ω* = *r*d*ϕ* d*y* is zero as *r* → 0. These cylindrical waves are evanescent and vanish in the limit *r* >> *λ*.
3. Equations (A3b) and (A4a,b) show that (*u*_S_ – *û*_S_) and ∂(*u*_S_ – *û*_S_)/∂*x* are continuous on crossing the screen (*ϕ* = 0 → *ϕ* = 2π), whereas ∂(*u*_S_ – *û*_S_)/∂*z* is not. Conversely, ∂(*u*_S_ + *û*_S_)/∂*z* is continuous, whereas (*u*_S_ – *û*_S_) and ∂(*u*_S_ + *û*_S_)/∂*x* are discontinuous. These are the expected properties of the tangential (magnetic or electric) field vector components when the light field is polarized parallel or perpendicular to the diffracting edge, and thus it is permissible to apply Maxwell’s equations in the form
  $$H^{\left(p\right)}=\sqrt{2E_{0}}4\sqrt{\frac{\varepsilon }{\mu }}(\mu_{S}-{\hat{u}}_{S})m,\;E^{\left(p\right)}=\frac{i}{k}\sqrt{\frac{\mu }{\varepsilon }}\text{curl}\;H^{\left(p\right)},$$ (A5a)
  $$E^{\left(s\right)}=\sqrt{2E_{0}}4\sqrt{\frac{\mu }{\varepsilon }}(\mu_{S}+{\hat{u}}_{S})m,\;H^{\left(s\right)}=-\frac{i}{k}\sqrt{\frac{\varepsilon }{\mu }}\text{curl}\;E^{\left(p\right)},$$ (A5b)
  where **m** = [0,1,0] is the unit vector in the direction of the positive *y*-axis, *ε* and *μ* are the dielectric constant and magnetic permeability of the medium of propagation, and normalization factors were used so that the squared field vectors have the dimension of irradiance [W/m^2^]. On substitution of the derivatives Eqs. (A4a,b) and re-arranging terms, this leads to
  $$\begin{array}{l} E^{\left(p\right)}=\sqrt{2E_{0}}\sqrt[{4}]{\frac{\mu }{\varepsilon }}\left\{-\sin\alpha (u_{S}+{\hat{u}}_{S}),\;0,-\cos\alpha (u_{S}+{\hat{u}}_{S})\right]\\ \frac{e^{i(kr-\frac{\pi }{4})}}{ik\sqrt{\lambda r}}[\sin\left(\frac{\phi }{2}+\frac{\alpha }{2}\right)-\sin\left(\frac{\phi }{2}-\frac{\alpha }{2}\right),\\ 0,\cos\left(\frac{\phi }{2}+\frac{\alpha }{2}\right)-\cos\left(\frac{\phi }{2}-\frac{\alpha }{2}\right)]\},\\ =\sqrt{2E_{0}}\sqrt[{4}]{\frac{\mu }{\varepsilon }}\left\{\left[u_{S}t+\frac{e^{i(kr-\frac{\pi }{4})}}{ik\sqrt{\lambda r}}v\right]-\left[{\hat{u}}_{S}\hat{t}+\frac{e^{i(kr-\frac{\pi }{4})}}{ik\sqrt{\lambda r}}\hat{v}\right]\right\} \end{array}$$ (A6a)
  $$\begin{array}{l} H^{\left(s\right)}=\sqrt{2E_{0}}\sqrt[{4}]{\frac{\varepsilon }{\mu }}\left\{-\sin\alpha (u_{S}+{\hat{u}}_{S}),\;0,-\cos\alpha (u_{S}-{\hat{u}}_{S})\right]\\ \frac{e^{i(kr-\frac{\pi }{4})}}{ik\sqrt{\lambda r}}[\sin\left(\frac{\phi }{2}+\frac{\alpha }{2}\right)+\sin\left(\frac{\phi }{2}-\frac{\alpha }{2}\right),\\ 0,\cos\left(\frac{\phi }{2}+\frac{\alpha }{2}\right)+\cos\left(\frac{\phi }{2}-\frac{\alpha }{2}\right)]\},\\ =\sqrt{2E_{0}}\sqrt[{4}]{\frac{\varepsilon }{\mu }}\left\{\left[u_{S}t+\frac{e^{i(kr-\frac{\pi }{4})}}{ik\sqrt{\lambda r}}v\right]+\left[{\hat{u}}_{S}\hat{t}+\frac{e^{i(kr-\frac{\pi }{4})}}{ik\sqrt{\lambda r}}\hat{v}\right]\right\} \end{array}$$ (A6b)
  where
  $$t=[-\sin\alpha ,0,-\cos\alpha ],\;\hat{t}=[\sin\alpha ,0,-\cos\alpha ]$$ (A6c)
  are the unit tangent vectors indicated in Fig. A1, and
  $$\begin{array}{l} v=\left[\sin\left(\frac{\phi }{2}+\frac{\alpha }{2}\right),0,\cos\left(\frac{\phi }{2}+\frac{\alpha }{2}\right)\right],\\ \hat{v}=\left[\sin\left(\frac{\phi }{2}-\frac{\alpha }{2}\right),0,\cos\left(\frac{\phi }{2}-\frac{\alpha }{2}\right)\right] \end{array}$$ (A6d)
  are unit vectors in the directions of the above-mentioned evanescent cylindrical waves. The middle portions of Eqs. (A6a) and (A6b) are equivalent to the expressions cited by Bouwkamp [11] for the electromagnetic field components given by Sommerfeld’s solution. However, it appears that their representation in terms of the unit vectors defined by Eqs. (A6c,d) has not previously appeared in the literature.
4. Equations (A5a,b) and (A6a,b) express each of the electromagnetic field vectors as the sum or difference of two components which, like Sommerfeld’s wave functions *u* and *û* themselves, are easily recognized as representative of a forward or reverse wave motion. It is obvious that these components must be mutually incoherent because, in all cases of practical interest, they propagate in opposite or nearly opposite directions, thus precluding any interference between them. Therefore, the corresponding Poynting vectors are given by the expressions
  $$E^{\left(p\right)}\times H^{(p)*}=2E_{0}\{[-u_{S}t+\frac{e^{i(kr-\frac{ð}{4}})}{ik\sqrt{\lambda r}}v]\times u_{S}^{*}m]-[-{\hat{u}}_{S}\hat{t}+\frac{e^{i(kr-\frac{ð}{4}})}{ik\sqrt{\lambda r}}\hat{v}]\times {\hat{u}}_{S}^{*}m]\}$$ (A7a)
  $$E^{\left(s\right)}\times H^{(s)*}=2E_{0}\{u_{S}m\times [u_{S}^{*}t-\frac{e^{-i(kr-\frac{ð}{4}})}{ik\sqrt{\lambda r}}v]+{\hat{u}}_{S}m\times [{\hat{u}}_{S}^{*}\hat{t}-\frac{e^{-i(kr-\frac{ð}{4}})}{ik\sqrt{\lambda r}}\hat{v}]\},$$ (A7b)
  where it must be also be taken into account that, as mentioned above, the terms in $1/\sqrt{r}$ have no energetic significance. Thus, these terms must be ignored and the following simple result is obtained for the observable Poynting vectors [5] of the field,
  <S>=12Re[E(p)×H(p)*]≡12Re[E(s)×H(s)*]=E0(|uS|2s+|u^S|2s^)=ESs+E^Ss^, (A7c)
  where *E*_S_ and *Ê*_S_ are the forward and reverse irradiances^5^ incident on area elements normal to the unit vectors in the directions of propagation of the incident and reflected field,
  $$\begin{array}{l} s=m\times t=[-\cos\alpha ,0,\sin\alpha ],\\ \hat{s}=m\times \hat{t}=[-\cos\alpha ,0,-\sin\alpha ], \end{array}$$ (A7d)
  as indicated in Fig. A1. These equations are not a part of Sommerfeld’s original theory and may be interpreted as follows:
  - In the vicinity of the diffracting edge the wave functions *u*_S_ and *û*_S_, and thus the forward and reverse irradiances *E*_S_ and *Ê*_S_ as well, are similar in magnitude. Therefore, light traveling in both directions is present on both sides of the screen except, in the Fresnel approximation where *û*_S_ is negligibly small. This is a significant departure from the classical formulation of Huygens’ principle, where a reverse flow of energy on the positive side of the aperture is precluded by the explicit assumption that light does not travel backwards. Instead, Sommerfeld’s theory asserts the presence of a bidirectional flow of energy on both sides of the aperture plane, and in this connection it is relevant to cite two earlier papers on Sommerfeld’s theory by Braunbek [12] and Braunbek and Laukien [13]. The latter includes an interesting diagram depicting a swirling, bidirectional flow of energy at sub-wavelength distances from the diffracting edge. Although it appears that Braunbek and Laukien assumed a coherent superposition of the forward and reverse fields, the eddy currents shown in their diagram can be regarded as Maxwellian analogues of Huygens’ wavelets.
  - In spite of the explicit assumption of separate boundary conditions for *p*- and *s*-polarized incident light, the forward and reverse irradiances *E*_S_ and *Ê*_S_ defined by Eq. (A7c) are the same in both cases. The composite wave functions *u*_S_ ∓ û_S_ pertain to different states of polarizations only insofar as their phases are concerned, but their squared magnitudes cannot be used to describe the energy flow in the field as they contain non-observable cross terms in *u*_S_*û*_S_. This distinction disappears in the Fresnel region, where the field on the positive side of the aperture plane is unidirectional (*û*_S_ << *u*_S_) and the usual definition of irradiance as the squared magnitude of the total wave function is justified.
5. For a normally incident field (*α* = π/2) one finds
  cos(ϕ∓α)=±sinϕ, (A8a)
  $$\begin{array}{l} \cos\left[\frac{1}{2}\left(\phi \mp \alpha \right)\right]=\frac{1}{\sqrt{2}}\left(\cos\frac{\phi }{2}\pm \sin\frac{\phi }{2}\right)\\ =\frac{1}{2}\left(\sqrt{1+\cos\phi }\pm \sqrt{1-\cos\phi }\right) \end{array}$$ (A8b)

Hence, the starting equations in Secs. 2 and 3 of the main text are obtained by using Eqs. (A1a) to reintroduce Cartesian coordinates and noting that the sine term in the central portion of Eq. (A8b) is always positive, while the cosine term and *z* are opposite in sign.

## 6. Appendix B. Wave-Equation Conformance

It is commonly agreed that one of the most important measures of the physical significance and mathematical rigor of scalar diffraction theories is whether, or how well, they satisfy the wave equation [Eq. (A2a)]. In this appendix, this aspect of the Sommerfeld and Rayleigh-Sommerfeld theories is analyzed. It is shown that Sommerfeld’s solution obeys the wave equation rigorously everywhere in space, whereas the Rayleigh-Sommerfeld integrals (and, thus, Kirchhoff’s integral as well) exhibit deviations from the wave equation in the immediate proximity of the aperture plane.

### 6.1 Sommerfeld’s Solution

In this subsection, Sommerfeld’s solution (A3a–d) will be written as

$$\begin{array}{l} u_{S}\;\text{or}\;{\hat{u}}_{S}=u=e^{ikr\;\cos\;\beta }V\left(\rho \right),\\ \rho \;\text{or}\;\hat{\rho }=\sqrt{\frac{8r}{\lambda }}\cos\left(\frac{1}{2}\beta \right),\;\beta =\phi \mp \alpha . \end{array}$$ (A9a)

Thus, ∂/∂*ϕ* = ∂/∂*β* and

$$\begin{array}{l} \frac{\partial V}{\partial r}=\frac{1}{\sqrt{\lambda r}}\cos\left(\frac{1}{2}\beta \right)e^{i\left[kr\left(1-\cos\beta \right)-\frac{ð}{4}\right]},\\ \frac{\partial V}{\partial \phi }=\frac{1}{\sqrt{\lambda r}}\cos\left(\frac{1}{2}\beta \right)e^{i\left[kr\left(1-\cos\beta \right)-\frac{ð}{4}\right]}, \end{array}$$ (A9b)

$$\frac{\partial u}{\partial r}=-ik\cos\beta u+\frac{1}{\sqrt{\lambda r}}\cos\left(\frac{1}{2}\beta \right)e^{i\left(kr-\frac{ð}{4}\right)},$$ (A9c)

$$\begin{array}{l} \frac{\partial^{2}u}{\partial r^{2}}=-k^{2}\cos^{2}\beta u\\ -\frac{1}{\sqrt{\lambda r}}\left[\frac{1}{2r}-2ik\sin^{2}\left(\frac{1}{2}\beta \right)\right]\cos\left(\frac{1}{2}\beta \right), \end{array}$$ (A9d)

$$\frac{\partial u}{\partial \phi }=ikr\;\sin\beta u-\sqrt{\frac{r}{\lambda }}\sin\left(\frac{1}{2}\beta \right)e^{i\left(kr-\frac{ð}{4}\right)},$$ (A9e)

$$\begin{array}{l} \frac{\partial^{2}u}{\partial \phi^{2}}=-\left(k^{2}r^{2}\sin^{2}\beta -ikr\cos\beta \right)u\\ -\frac{1}{\sqrt{\lambda r}}\left[\frac{r}{2}+2ikr^{2}\sin^{2}\left(\frac{1}{2}\beta \right)\right]\cos\left(\frac{1}{2}\beta \right)e^{i\left(kr-\frac{ð}{4}\right)}. \end{array}$$ (A9f)

Hence it follows immediately that the wave equation, Eq. (A2a), is rigorously satisfied. It should be noted that this is true everywhere in space, even at the diffracting edge itself where each component of the Laplace operator is singular.

### 6.2 Rayleigh-Sommerfeld Integrals

In this next subsection, the wave equation conformance of the Rayleigh-Sommerfeld integrals $u_{\text{RS}}^{\left(p,s\right)}(x,\;z)$ is analyzed by computing numerical values of the quantities,

$$w_{\text{RS}}^{\left(p,s\right)}\left(x,\;z\right)=\left[\frac{1}{k^{2}}\left(\frac{\partial^{2}}{\partial x^{2}}+\frac{\partial^{2}}{\partial z^{2}}\right)+1\right]u_{\text{RS}}^{\left(p,s\right)}\left(x,\;z\right),$$ (A10a)

which would everywhere be identically equal to zero if the wave equation is satisfied. For this purpose, the first and second derivatives of the Hankel functions appearing in Eqs. (2a,2b) are evaluated by means of the identities

$$\frac{{\text{dH}}_{0}^{\left(1\right)}\left(\beta \right)}{d\beta }=-H_{1}^{\left(1\right)}\left(\beta \right),\;\frac{d}{d\beta }\left[\frac{H_{1}^{\left(1\right)}\left(\beta \right)}{\beta }\right]=-\frac{H_{2}^{\left(1\right)}\left(\beta \right)}{\beta },$$ (A10b)

$$\frac{d}{d\beta }\left[\frac{H_{2}^{\left(1\right)}\left(\beta \right)}{\beta^{2}}\right]=-\frac{H_{3}^{\left(1\right)}\left(\beta \right)}{\beta^{2}},\frac{\partial \beta }{\partial x}=\frac{k^{2}x}{\beta },\frac{\partial \beta }{\partial z}=\frac{k^{2}z}{\beta }.$$ (A10c)

Hence, the required *x*-derivatives of $u_{\text{RS}}^{\left(p,s\right)}$ are found by differentiating Eqs. (2a,b) with respect to the upper limit of integration, yielding

$$\begin{array}{c} \frac{\partial u_{\text{RS}}^{\left(p\right)}}{\partial x}=\frac{kH_{0}^{\left(1\right)}\left(\beta_{x}\right)}{2},\;\frac{\partial u_{\text{RS}}^{\left(s\right)}}{\partial x}=\frac{ik^{2}zH_{1}^{\left(1\right)}\left(\beta_{x}\right)}{2\beta_{x}},\\ \beta_{x}=k\sqrt{x^{2}+z^{2}}, \end{array}$$ (A10d)

$$\frac{\partial^{2}u_{\text{RS}}^{\left(p\right)}}{\partial x^{2}}=-\frac{k^{3}xH_{1}^{\left(1\right)}\left(\beta_{x}\right)}{\beta_{x}},\;\frac{\partial^{2}u_{\text{RS}}^{\left(s\right)}}{\partial x^{2}}=-\frac{ik^{4}\;xzH_{2}^{\left(1\right)}\left(\beta_{x}\right)}{2\beta_{x}^{2}},$$ (A10e)

and the *z*-derivatives are obtained by substitution of

$$\frac{\partial^{2}H_{0}^{\left(1\right)}\left(\beta \right)}{\partial z^{2}}=-k^{2}\left[\frac{H_{1}^{\left(1\right)}\left(\beta \right)}{\beta }-\frac{{\left(kz\right)}^{2}H_{2}^{\left(1\right)}\left(\beta \right)}{\beta^{2}}\right],$$ (A10f)

$$\frac{\partial^{2}}{\partial z^{2}}\left[\frac{ik^{2}zH_{1}^{\left(1\right)}\left(\beta \right)}{2\beta }\right]=-\frac{ik^{4}z}{2}\left[\frac{{3H}_{2}^{\left(1\right)}\left(\beta \right)}{\beta^{2}}-\frac{{\left(kz\right)}^{2}H_{3}^{\left(1\right)}\left(\beta \right)}{\beta^{3}}\right],$$ (A10g)

into Eqs. (2a,b). The final expressions are

$$\begin{array}{l} w_{\text{RS}}^{\left(p\right)}\left(x,\;z\right)=-\frac{kxH_{1}^{\left(1\right)}\left(\beta_{x}\right)}{\beta_{x}}\\ +\frac{k}{2}\int_{-\infty }^{-x}d\left(\xi -x\right)\left[H_{0}^{\left(1\right)}\left(\beta \right)-\frac{H_{1}^{\left(1\right)}\left(\beta \right)}{\beta }+\frac{{\left(kz\right)}^{2}H_{2}^{\left(1\right)}\left(\beta \right)}{\beta^{2}}\right], \end{array}$$ (A10h)

$$\begin{array}{l} w_{\text{RS}}^{\left(s\right)}\left(x,\;z\right)=-\frac{ik^{2}xzH_{2}^{\left(1\right)}\left(\beta_{x}\right)}{\beta_{x}^{2}}\\ +\frac{ik^{2}z}{2}\int_{-\infty }^{-x}d\left(\xi -x\right)\left[\frac{H_{1}^{\left(1\right)}\left(\beta \right)}{\beta }-\frac{3H_{2}^{\left(1\right)}\left(\beta \right)}{\beta^{2}}+\frac{{\left(kz\right)}^{2}H_{3}^{\left(1\right)}\left(\beta \right)}{\beta^{3}}\right]. \end{array}$$ (A10i)

The integrals on the right-hand side of these expressions were evaluated by recursive numerical integration as described in Ref. [7]. In accordance with Eq. (3b), the starting values used for these computations were $w_{\text{RS}}^{\left(p,s\right)}(0,\;z)=0$.

The real and imaginary parts of (A10h,i) are plotted in Fig. A2 for *z* = 0.1, showing that at this distance the wave-equation failure is substantial. Additional computations indicated that the corresponding values of $w_{\text{RS}}^{\left(p,s\right)}(x,\;z)$ decrease at larger distances *z*, but fall below 0.01 only when *z* > 30*λ* so that calculable departures from the wave equation are present throughout the near zone. This is not surprising because, otherwise, the Rayleigh-Sommerfeld and Kirchhoff boundary-value integrals would be rigorously correct. On the other hand, the above results are at odds with a fallacious belief that these integrals must obey the wave equation because their integrands do. The fact of the matter is that the assumed boundary conditions Eqs. (2c,d) abruptly truncate the incident field at the edge and, thus, transform these integrands into discontinuous functions that violate the wave equation.
